# Supplementary material for: Predicting trajectories of illness using RNA velocity of whole blood
Source: Nat Commun. 2026 May 6;17:3652. doi: 10.1038/s41467-026-71685-5 (PMC13149583; doi:10.1038/s41467-026-71685-5)
Supplement: Supplementary file 1 — Supplementary Information [file 41467_2026_71685_MOESM1_ESM.pdf]

Predicting trajectories of illness using RNA velocity of whole blood

Supplementary Figures and Tables

Supplementary Table 1: Sample numbers and mapping statistics for RNA-Seq datasets.

Dataset	Number of samples, subjects	Uniquely mapped reads (% total reads)	Quantified reads, % range (mean)
Influenza CHIM	n=138 from n=23 individuals	Mean: 24,381,075 (92.5%)<br>Range: 18,566,465-29,205,267 (87.5-95.6%)	Exonic: 37.0-53.6 (42.7)<br>Intronic: 44.2-57.4 (50.8)
SARS-CoV-2 CHIM	n=257 from n=26 individuals	Mean: 57,575,110 (82.4%)<br>Range: 23,776,992-128,131,106 (74.0-90.3%)	Exonic: 33.3-51.3 (40.0)<br>Intronic: 41.8-62.2 (53.9)
INSTINCT	n=138 from 56 individuals	Mean: 16,064,943 (61.3%)<br>Range: 7,654,859-40,798,582 (33.1-83.1%)	Exonic: 20.3-68.5 (56.5)<br>Intronic: 28.4-46.3 (37.3)
TB-IRIS	n=95 individuals	Mean: 33,645,255 (79.2%)<br>Range: 22,624,044-55,714,568 (57.9-89.1%)	Exonic: 36.6-62.4 (43.4)<br>Intronic: 29.6-56.7 (49.3)
PERFORM	n=418 from n=416 individuals	Mean: 18,229,656 (94.5%)<br>Range: 2,991,328-79,250,659 (83.0-95.8%)	Exonic: 36.2-78.2 (43.9)<br>Intronic: 6.5-60.7 (51.0)
IBD	n=24 from n=13 individuals	Mean: 27,398,292 (72.7%)<br>Range: 15,617,445-40,099,173 (61.4-84.8%)	Exonic: 82.9-94.7 (89.9)<br>Intronic: 11.1-18.7 (13.8)

Abbreviations: Controlled Human Infection Model (CHIM), Integrated Network for Surveillance, Trials and Investigations into COVID-19 Transmission (INSTINCT), Tuberculosis (TB)-associated Immune Reconstitution Inflammatory Syndrome (TB-IRIS), Personalized Risk assessment in Febrile illness to Optimize Real-life Management (PERFORM), Inflammatory Bowel Disease (IBD)

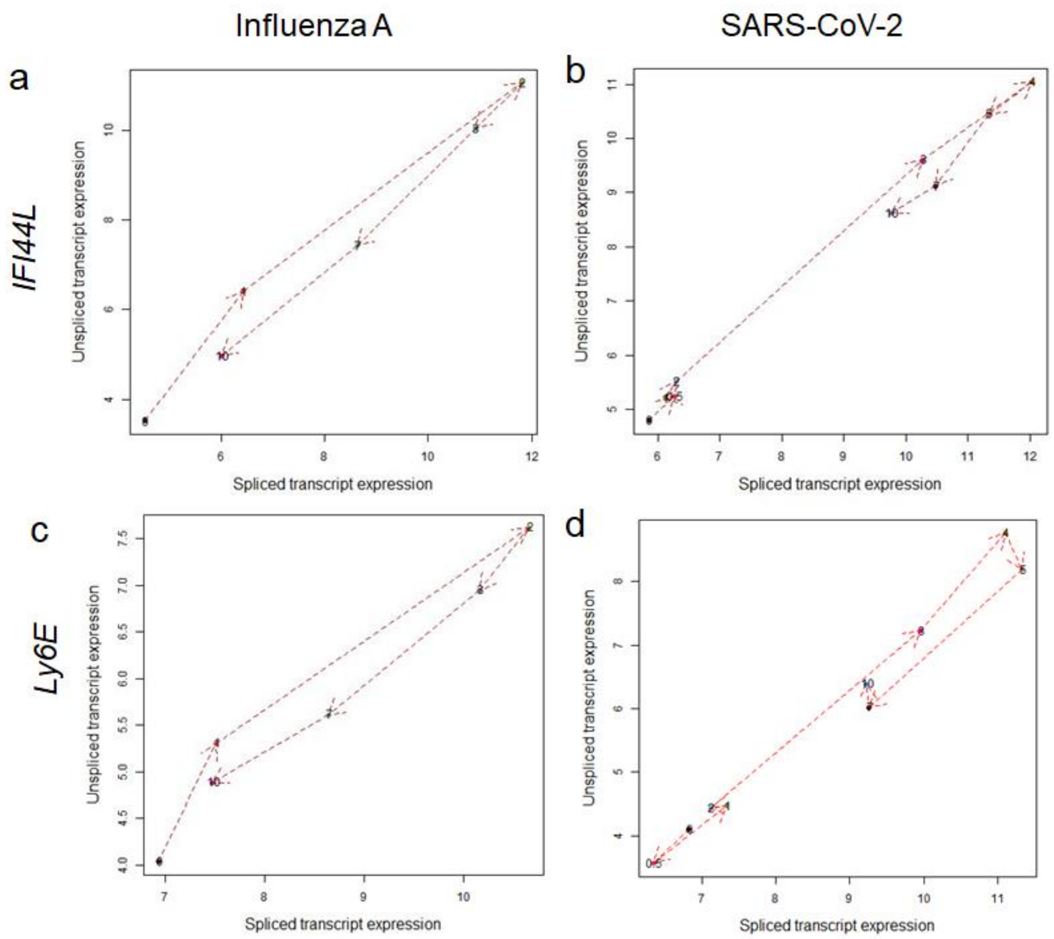

Supplementary Fig. 1: Phase portraits of the spliced and unspliced transcript expression of Interferon Induced Protein 44-Like (*IFI44L*) and Lymphocyte antigen 6E (*Ly6E*) in both controlled human infection model datasets. Samples from the subject ID SPFC019 were used to generate the plots from the influenza A dataset (n=6 samples). Samples from subject ID 635729 were used to generate the plots from the SARS-CoV-2 dataset (n=9 samples). Each sample point is coloured by its corresponding time-point. Arrows point in the direction of time. Source data are provided as a Source Data file.

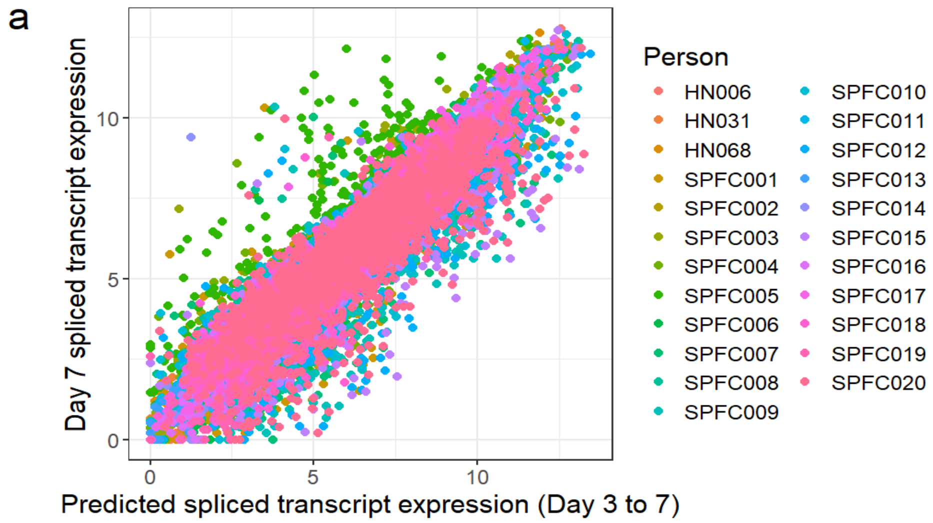

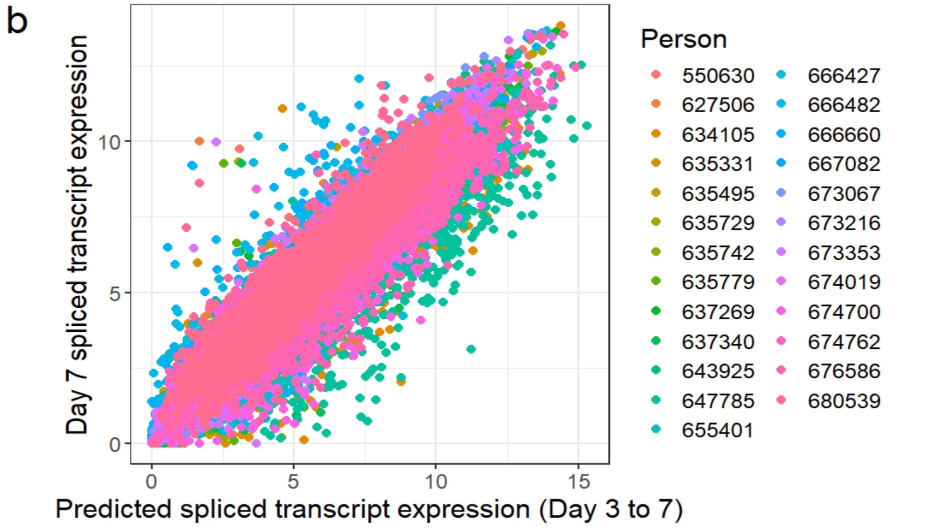

Supplementary Fig. 2: The actual spliced transcript expression at day 7 versus the predicted values from day 3 in the Influenza A (a) and SARS-CoV-2 controlled human infection model datasets.

a | The predicted future spliced transcript expression (calculated from day 3) versus actual spliced transcript expression at day 7 for 1,563 genes (points) selected using maSigPro (colored by subject sample ID, n=23 subjects).

b| The predicted future spliced transcript expression (calculated from day 3) versus the actual spliced transcript expression at day 7 for 2,700 genes (points) selected using maSigPro (colored by sample subject ID, n=25 subjects, one subject did not have a day 7 RNA-Seq sample available).

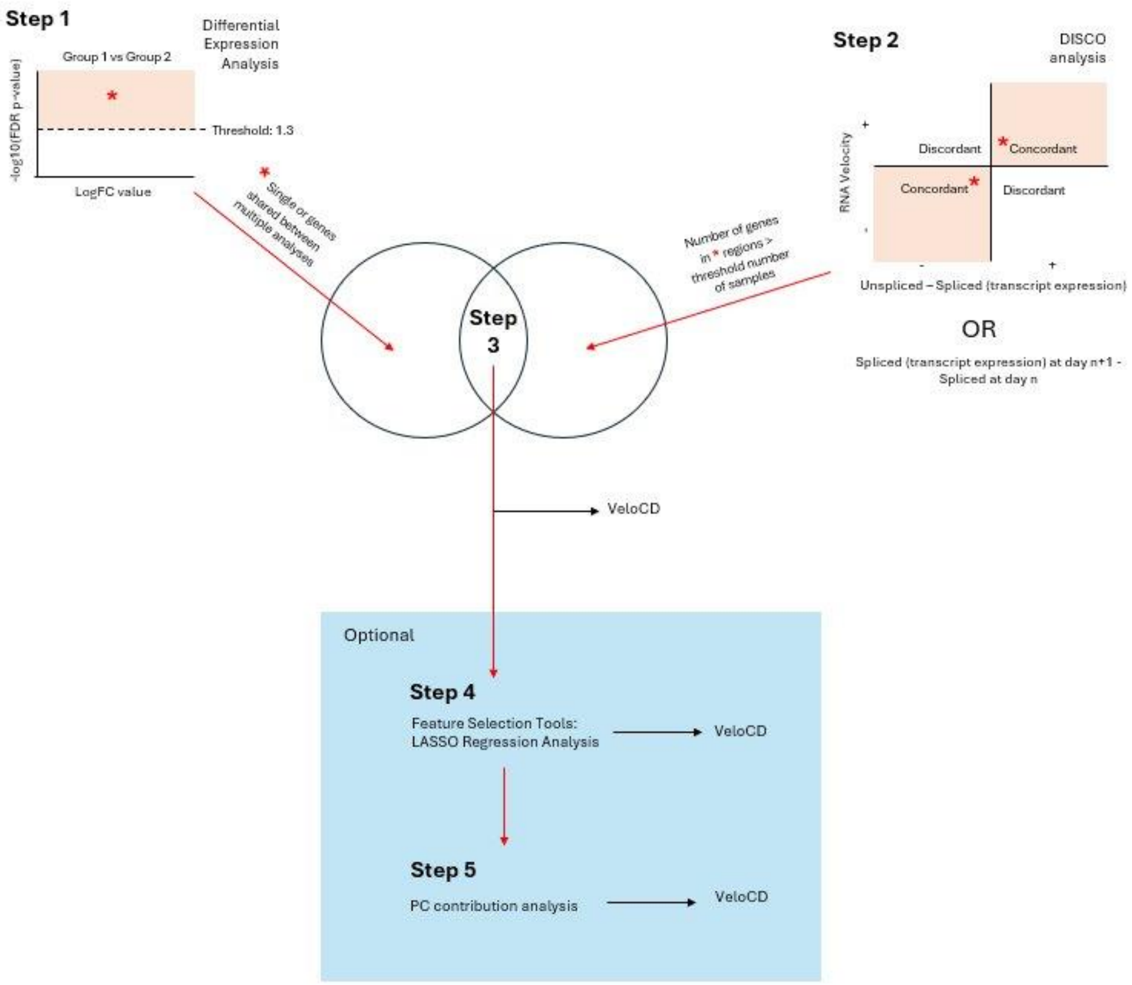

Supplementary Fig. 3: The general framework for selecting genes suitable for RNA velocity-based prognosis prediction using VeloCD.

The first three steps are non-optional, steps 4 and 5 are optional if a minimal gene signature is required and can include the use of feature selection tools such as Least Absolute Shrinkage and Selection Operator (LASSO) regression and selection of genes contributing the most variation to a user-specified number of principal components. Step 1 is differential expression analysis between reference groups, with a user-selected False Discovery Rate (FDR) p-value threshold. Step 2 is Discordance-Concordance (DISCO) analysis of each gene's RNA velocity value (y-axis) vs either its unspliced minus spliced transcript abundance or its change in spliced transcript expression over the timescale of interest (day n+1). Genes with concordance of both metrics in at least a user-selected number of samples are taken forward for further analysis. The intersection of genes selected in steps 1 and 2 is calculated, and these are taken forward as the input for VeloCD.

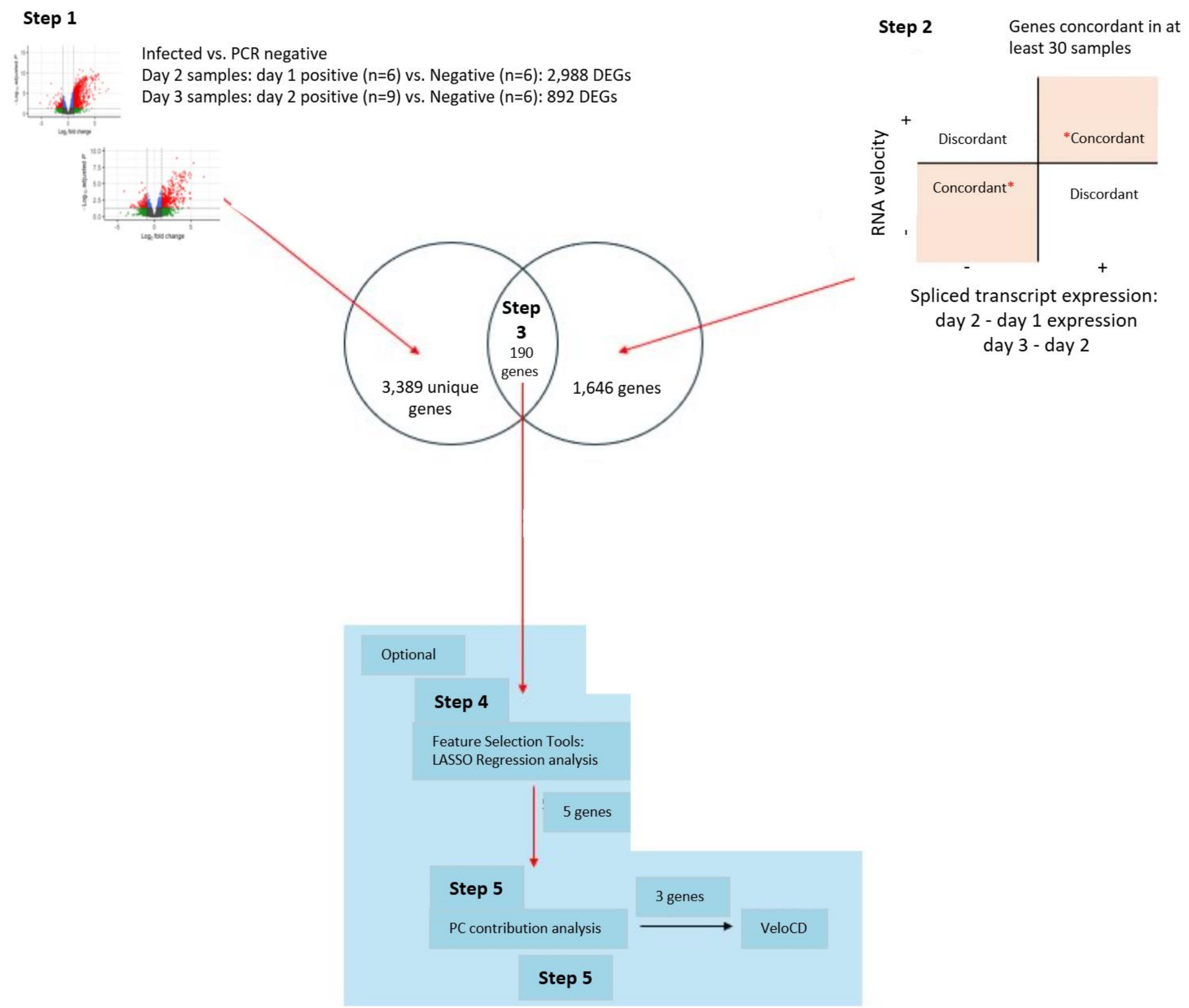

Supplementary Fig. 4: The application of the gene selection framework to the influenza A CHIM RNA-Seq dataset. Step 1 illustrates the results of the DEA between the infected subjects and subjects who remain Polymerase Chain Reaction (PCR) negative throughout the study (stratified by the day the subject first tested positive). Step 2 illustrates the Discordance-Concordance analysis of the day 1, 2 and 3 samples from this cohort. Step 3 shows the number of genes intersecting between these two analyses. These were then put through Least Absolute Shrinkage and Selection Operator (LASSO) regression (step 4) and the genes that contribute the most to the top 3 Principal Components (PCs, step 5) were used for RNA velocity analysis.

Supplementary Table 2: The contribution (%) of each gene in the influenza A signature to the first three principal components (PCs) of a PCA constructed using the reference set of samples (n=22) and five genes.

Gene Symbol	PC1 Contribution (%)	PC2 Contribution (%)	PC3 Contribution (%)
SLC9A3-AS1	51.1	46.2	0.32
BAK1	27.1	35.8	25.8
APOL3	9.95	14.1	8.09

Abbreviations: BCL2 Antagonist/Killer 1 (*BAK1*), SLC9A3 Antisense RNA 1 (*SLC9A3-AS1*), Apolipoprotein L3 (*APOL3*).

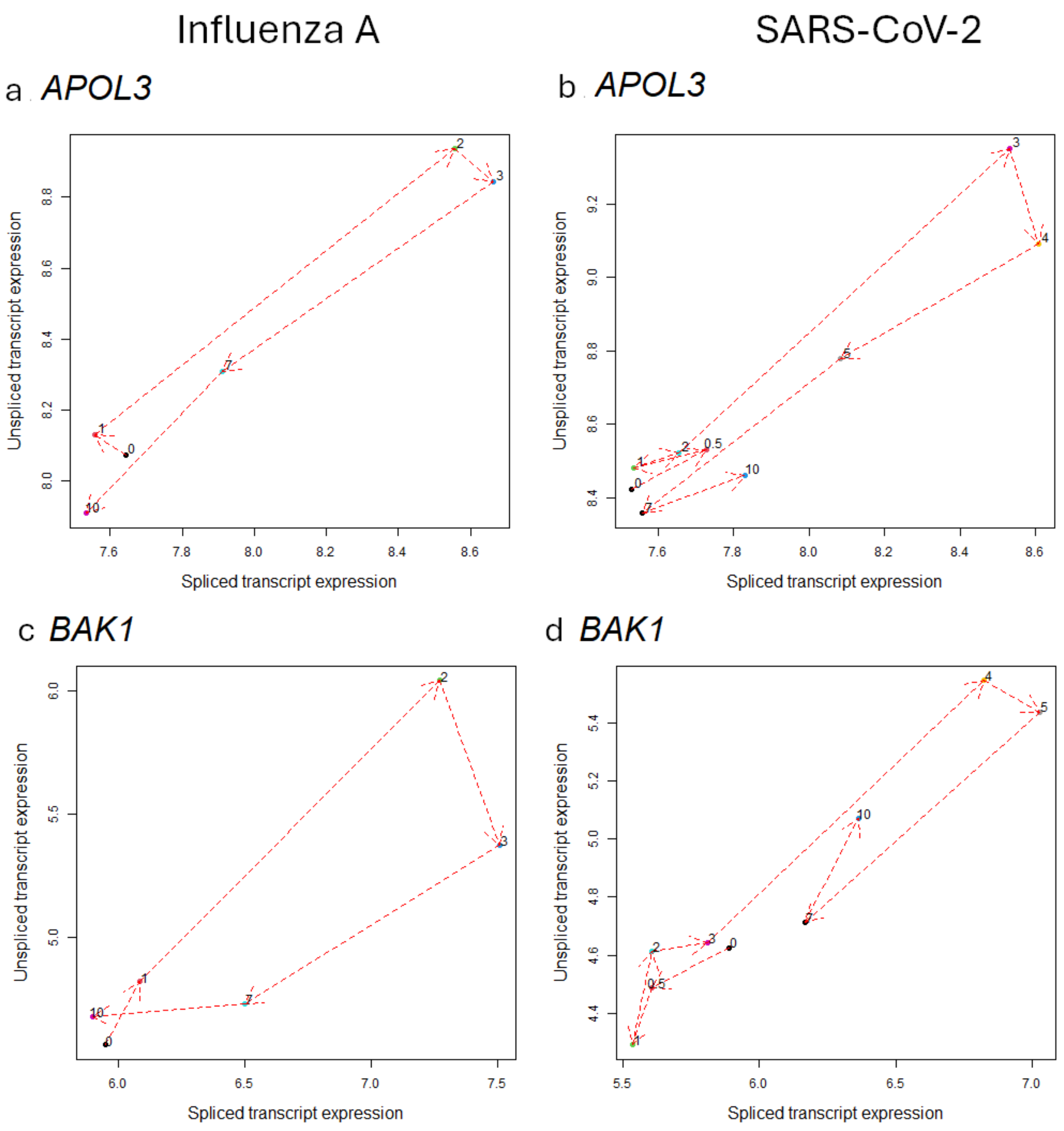

Supplementary Fig. 5: Phase Portraits of spliced and unspliced Apolipoprotein L3 (*APOL3*) and BCL2 Antagonist/Killer 1 (*BAK1*) mRNA expression during influenza A and SARS-CoV-2 infection.

a-d | Representative plots from single individuals showing the expression dynamics of *APOL3* (**a**, **b**) and *BAK1* (**c, d**) in the influenza A (**a, c,** n=6 samples, SPFC018 participant for *APOL3* and SPFC001 participant for *BAK1*) and SARS-CoV-2 (**b, d,** n=9 samples, participant 634105 for *APOL3* and participant 680539 for *BAK1*) cohorts. Timepoint 0.5 represents the post-inoculation day 0 time-point. Each sample point is coloured by its corresponding time-point. Arrows point in the direction of time. Source data are provided as a Source Data file.

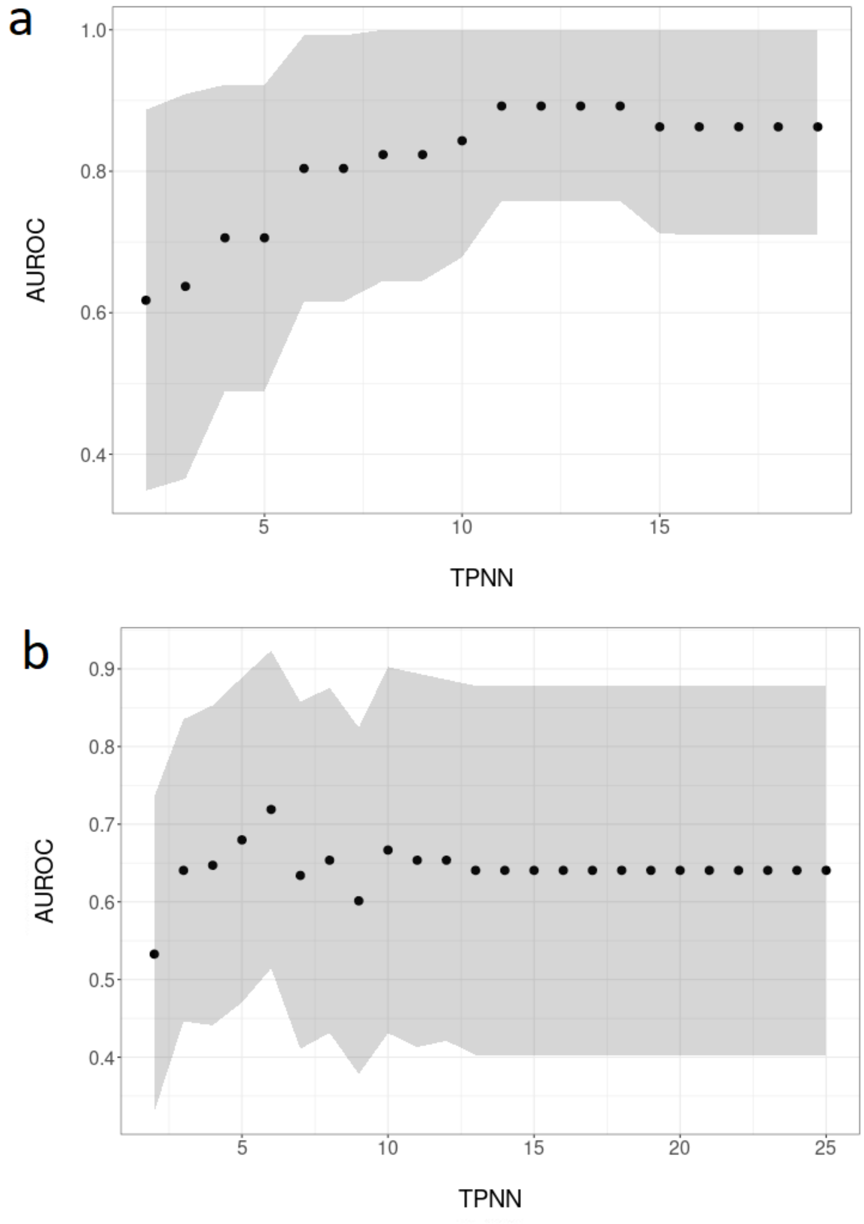

Supplementary Fig. 6: The range of Areas Under the Receiver Operating Characteristic (AUROC) curve values between Transition Probability Number of Neighbour (TPNN) runs for the controlled human infection model RNA-Seq datasets. Scatterplots showing the distribution of AUROC (shading represents 95% confidence intervals, distinguishing Polymerase Chain Reaction negative versus infected individuals) values across a range of TPNN values. **a** | influenza A (generated from n=23 subjects). **b** | SARS-CoV-2 (generated from n=26 subjects). Source data are provided as a Source Data file.

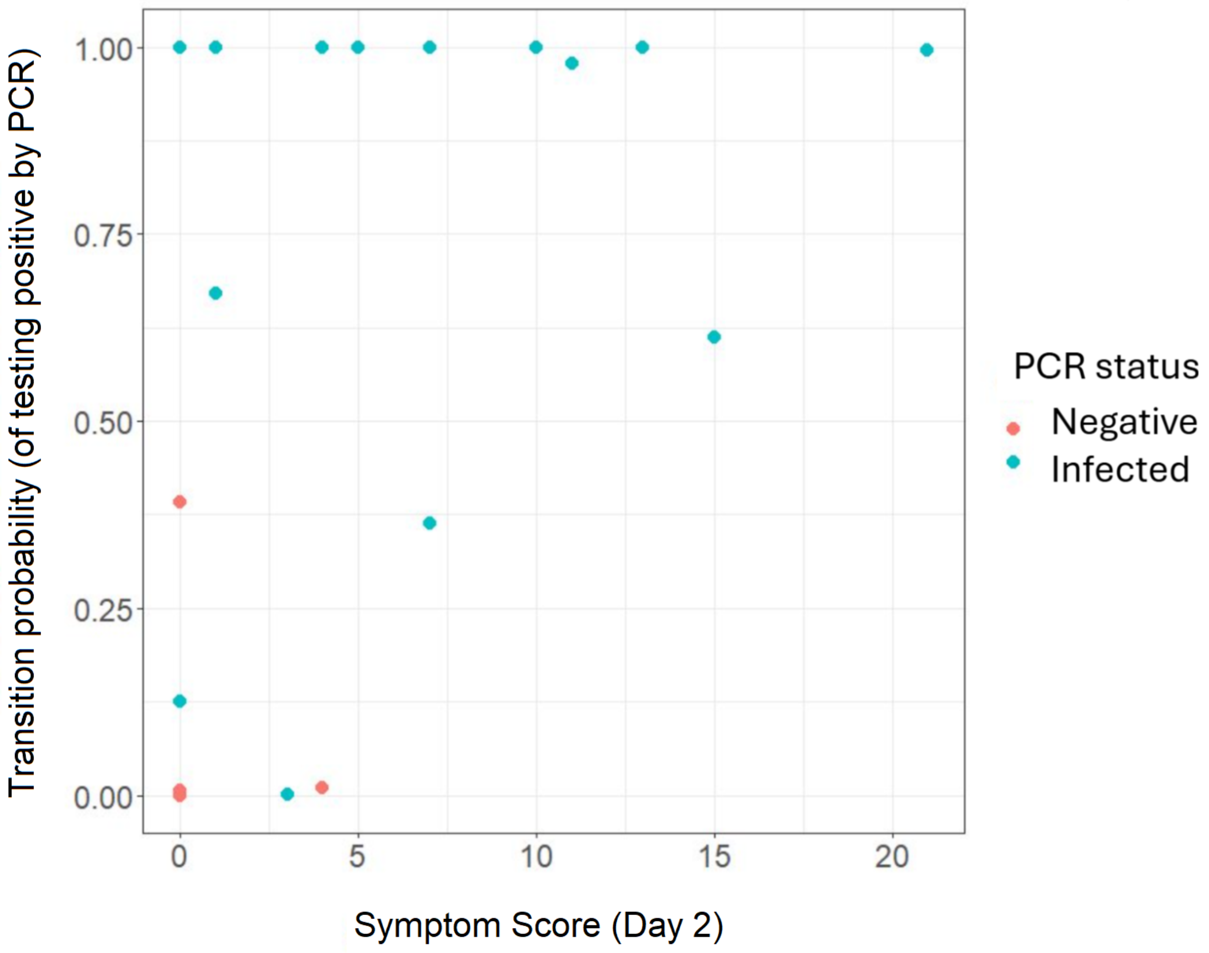

Supplementary Fig. 7: The relationship between the transition probability of testing positive by Polymerase Chain Reaction (PCR)-based tests calculated using day 1 samples and symptom scores measured at day 2 for the influenza controlled human infection model dataset (n=23 subjects). Points are coloured by PCR status (red: PCR negative, blue: infected). Source data are provided as a Source Data file.

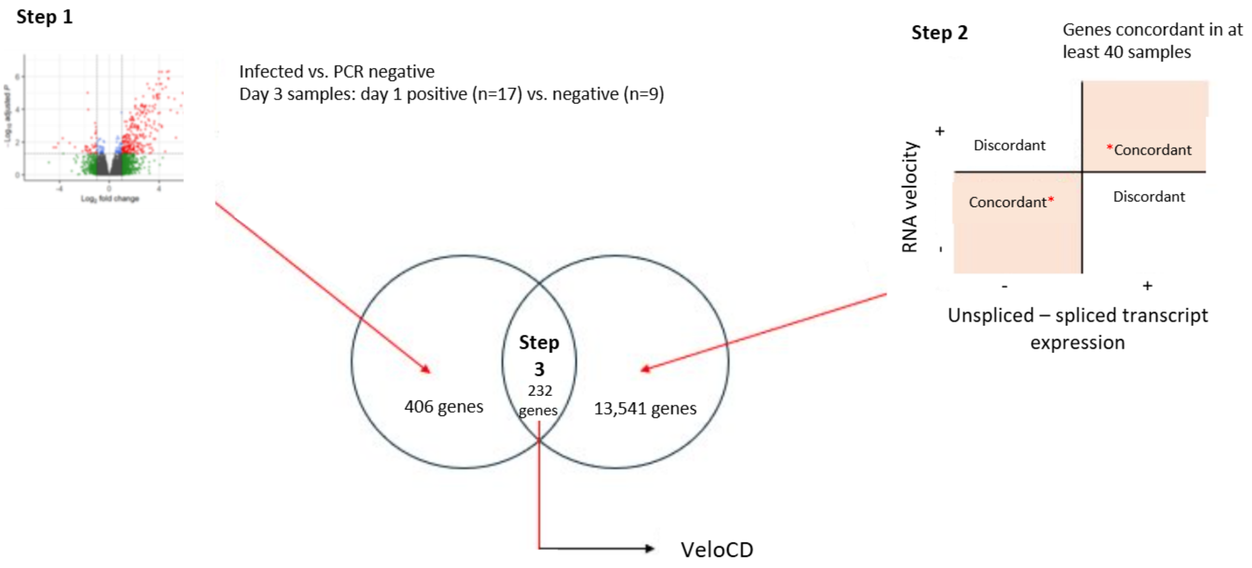

Supplementary Fig. 8: The application of the gene selection framework on the SARS-CoV-2 CHIM RNA-Seq dataset. Step 1 illustrates the results of the differential expression analysis between the infected subjects and subjects who remain Polymerase Chain Reaction (PCR)-negative throughout the study. Step 2 illustrates the Discordance-Concordance analysis of the day 0 (post-inoculation), 1, 2 and 3 samples from this cohort. Step 3 shows the number of genes intersecting between these two analyses, these were used for RNA velocity analysis.

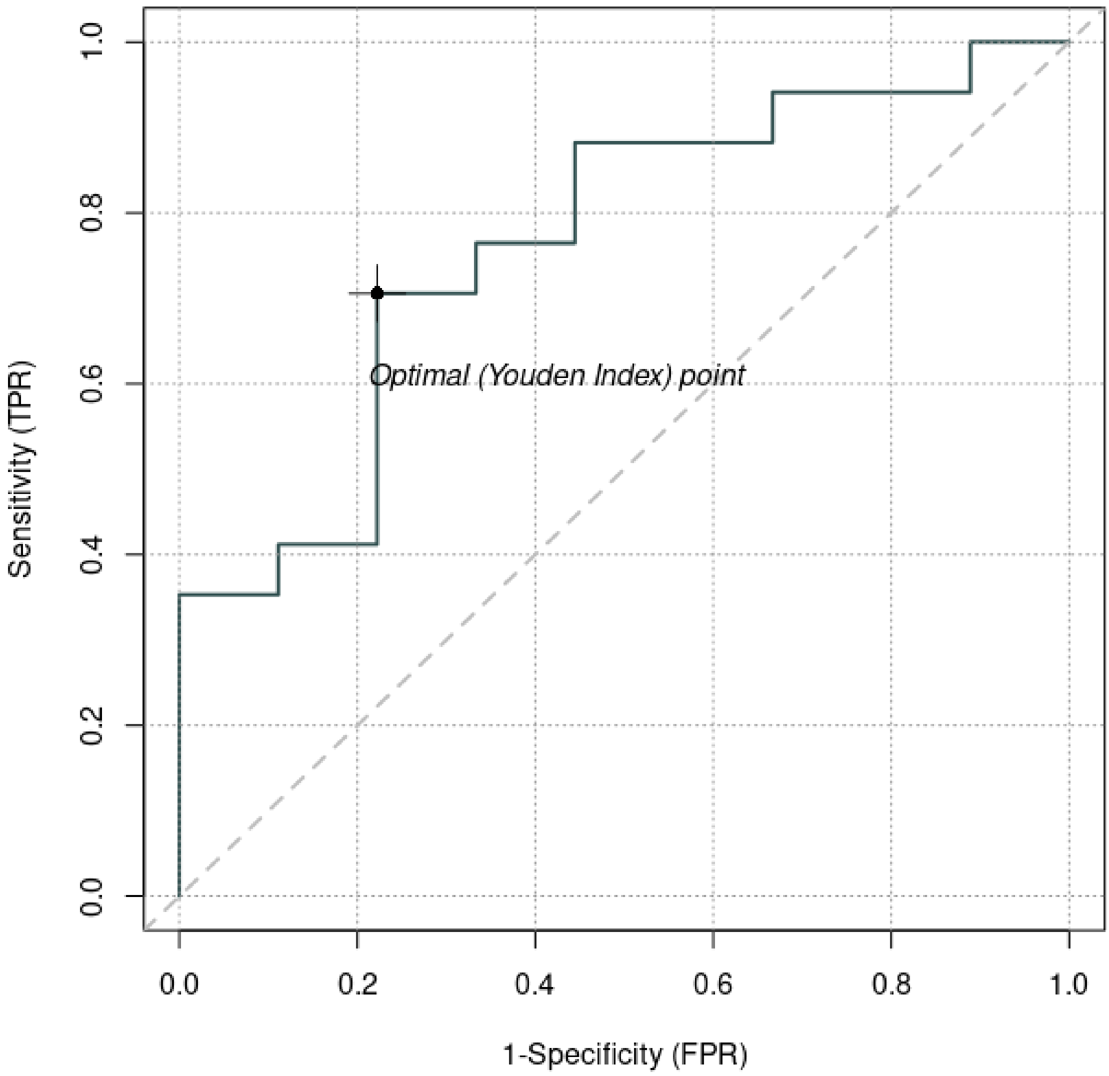

Supplementary Fig. 9: Performance of the influenza derived 3-gene signature for predicting future disease onset from day 1 post-inoculation in the SARS-CoV-2 controlled human infection model cohort (n=26 subjects, transition probability number of neighbours: 16, Principal Component Analysis-based fate maps). Area Under the Receiver Operating Characteristic curve is 0.76 (95% confidence interval: 0.57-0.96). Source data are provided as a Source Data file.

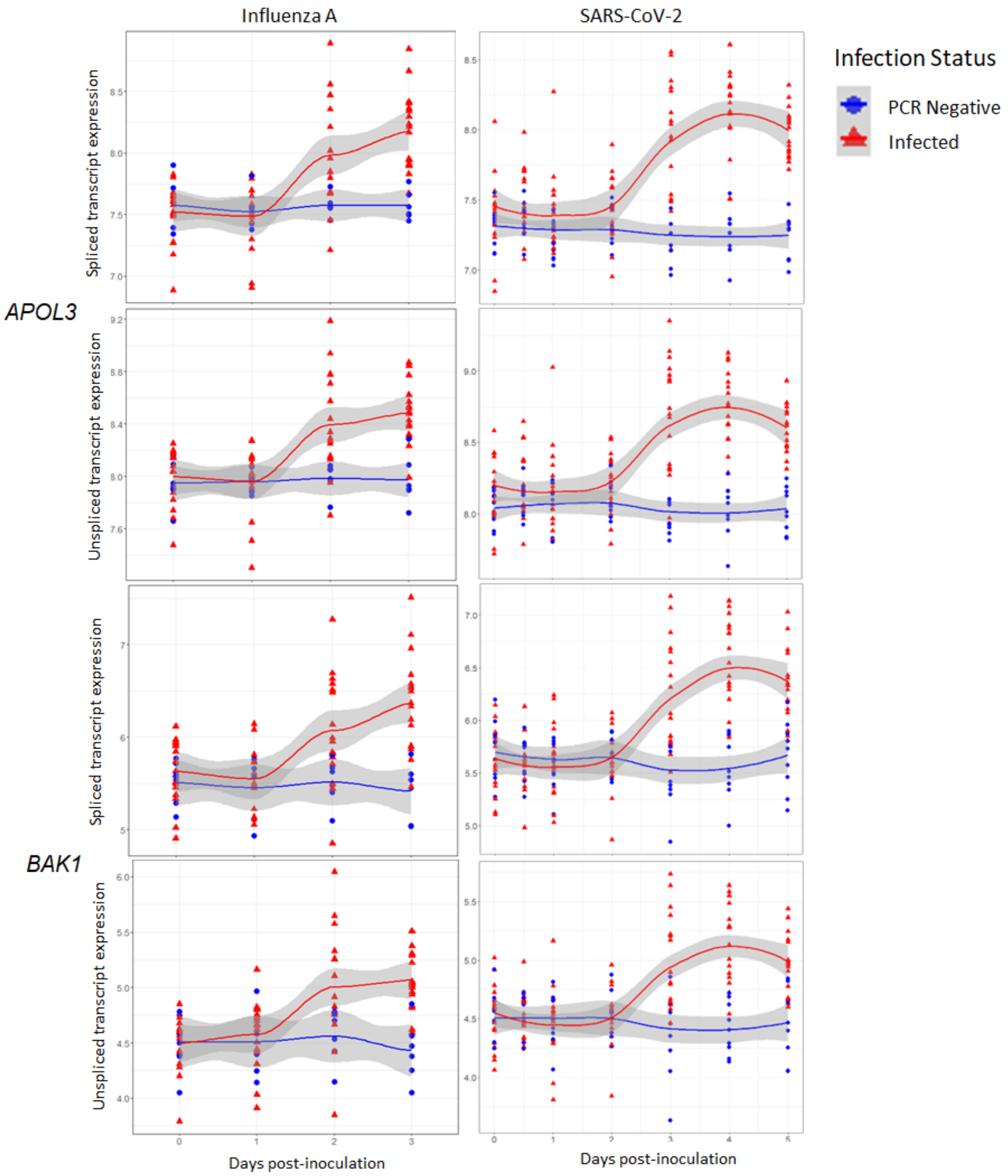

Supplementary Fig. 10: The temporal expression of spliced and unspliced transcripts of Apolipoprotein L3 *(APOL3)* and BCL2 Antagonist/Killer 1 (*BAK1*)

The spliced and unsliced transcript expression of *APOL3* and *BAK1* over time in the influenza (n=92; n=68 samples from the infected subjects, n=24 from the Polymerase Chain Reaction-PCR negative subjects) and SARS-CoV-2 (n=180 samples; n=117 from the infected subjects, n=63 from the PCR negative subjects) studies, showing infected (red triangles) and PCR negative subjects (blue circles). Time-point 0.5 represents the day 0 post-inoculation samples. Shading around the locally estimated scatterplot smoothing regression lines represents 95% confidence intervals. Source data are provided as a Source Data file.

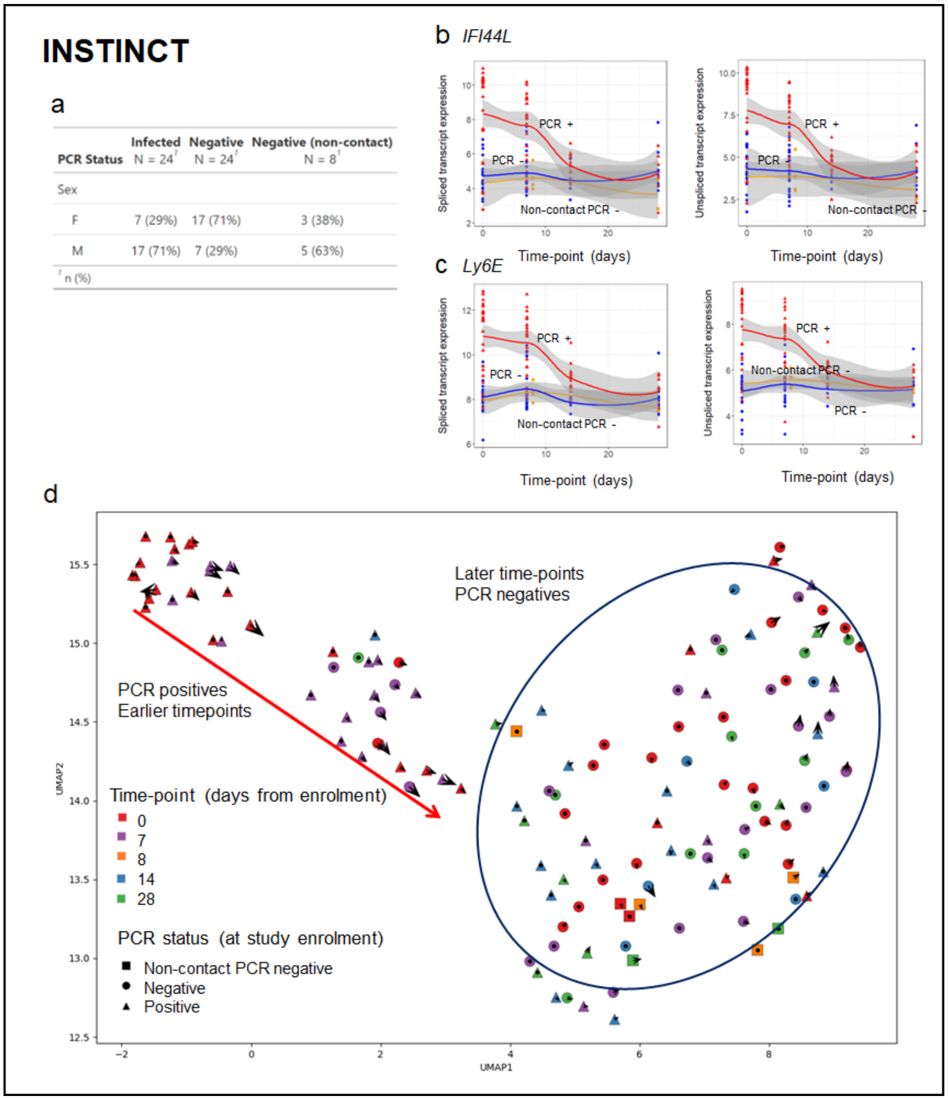

Supplementary Fig. 11: RNA velocity analysis of the Integrated Network for Surveillance, Trials and Investigations into COVID-19 Transmission (INSTINCT) study RNA-Seq dataset.

a | Characteristics of study subjects. **b** and **c** | spliced transcript expression of Interferon Induced Protein 44-Like gene (*IFI44L,* **b**) and Lymphocyte antigen 6E (*Ly6E,* **c**), genes (n=138 samples) with greatest changes over time between infected subjects (red triangles) and Polymerase Chain Reaction (PCR)-negative (blue circles) and non-contact PCR-negative subjects (yellow squares). Shading represents 95% confidence intervals around the locally estimated scatterplot smoothing regression lines. **d** | RNA velocity fate map (n=138 samples) of the 232 gene-signature from the previous SARS-CoV-2 applied to the INSTINCT cohort (number of neighbours: 25, transition probability number of neighbours: 25, Uniform Manifold Approximation and Projection-UMAP embedding method). The blue circle indicates the location of the PCR-negative subjects, and red arrow indicates trajectories of the infected individuals over time. Sample points are colored by timepoint and shaped by PCR-status at the time of study enrolment. Arrows on each sample correspond to RNA velocity values. Source data are provided as a Source Data file.

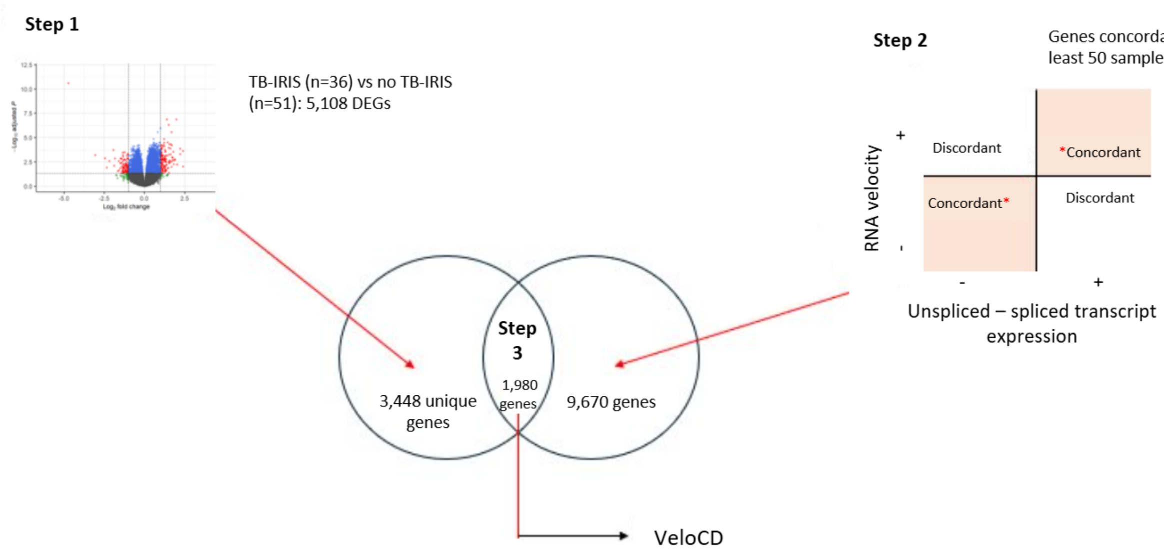

Supplementary Fig. 12: The application of the gene selection framework to the Tuberculosis (TB)-associated Immune Reconstitution Inflammatory Syndrome (TB-IRIS) study RNA-Seq dataset.

Step 1 illustrates the results of the differentially expression analysis between the subjects who developed TB-IRIS by the time of sampling and those who never developed the syndrome. Step 2 illustrates the Discordance-Concordance analysis of this dataset, which compared the direction of the RNA velocity and unspliced minus spliced (transcript expression). Step 3 shows the number of genes intersecting between these two analyses, these were used for RNA velocity analysis.

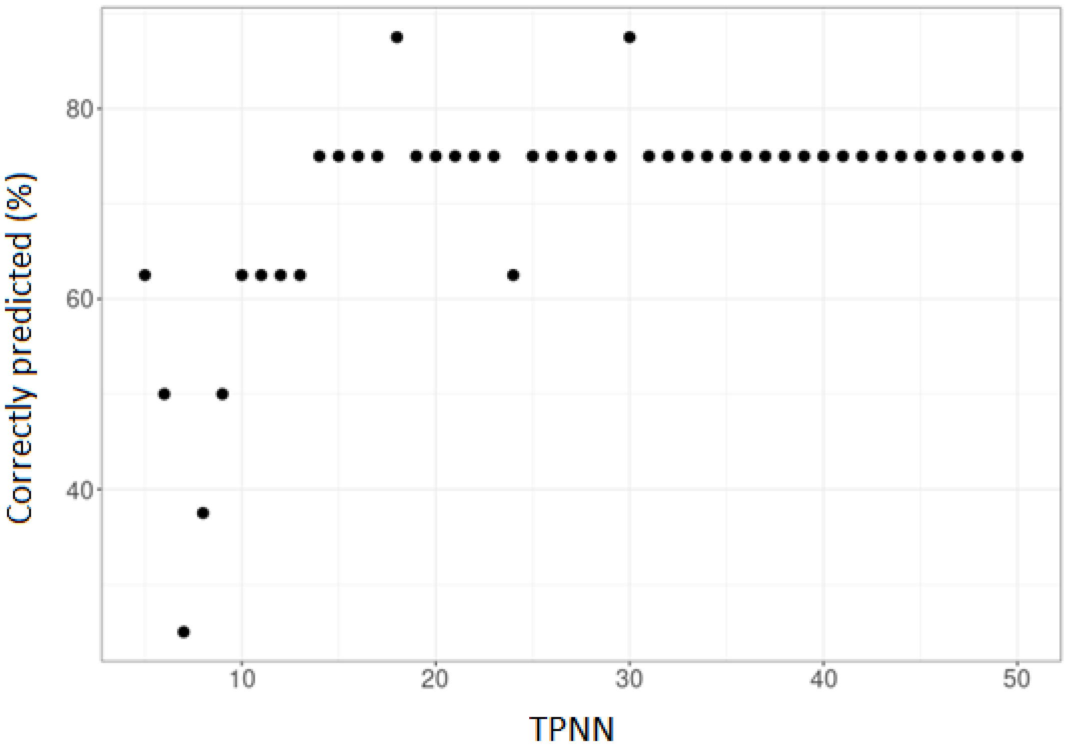

Supplementary Fig. 13: The relationship between the number of study subjects correctly predicted to develop Tuberculosis (TB)-associated Immune Reconstitution Inflammatory Syndrome (TB-IRIS) in the future and the Transition Probability Number of Neighbours (TPNN) value in the TB-IRIS study RNA-Seq dataset.

The percentage of the study subjects (n=8) correctly predicted to develop TB-IRIS in the future across a range of TPNN values. Source data are provided as a Source Data file.

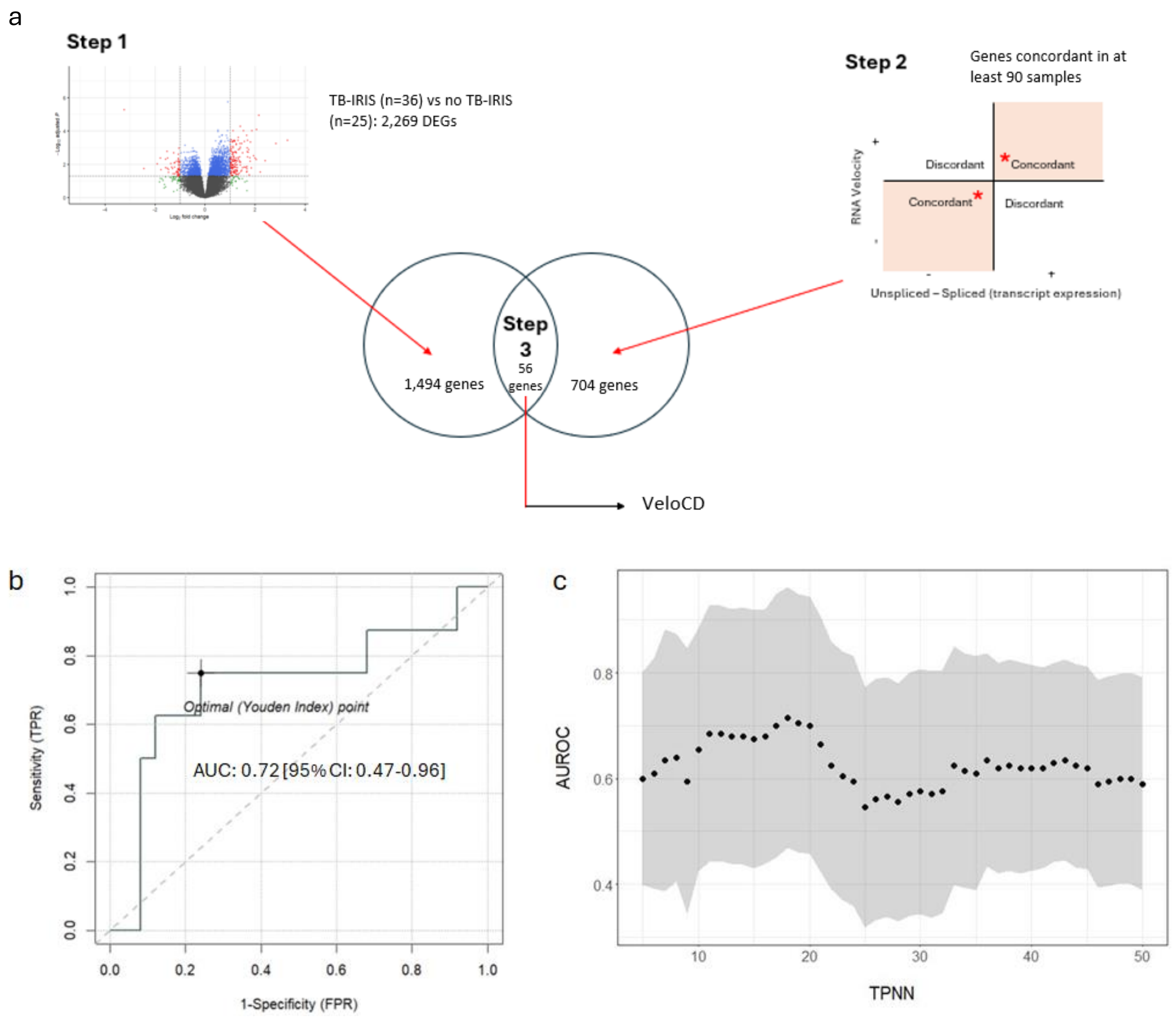

Supplementary Fig. 14: The predictive performance of RNA Velocity on the Tuberculosis (TB)-associated Immune Reconstitution Inflammatory Syndrome (TB-IRIS) dataset.

a | Gene selection criteria for this 56-gene signature. The X Inactive Specific Transcript (*XIST*) gene was removed from the full set of 2,269. 1,494 is the number of differentially expressed genes with spliced and unspliced transcript expression. **b** | A Receiver Operating Characteristic (ROC) curve of a 56-gene signature to predict future TB-IRIS status (positive, n=8, or remaining negative, n=25 subjects) in the TB-IRIS dataset (Transition Probability Number of Neighbours (TPNN): 18; number of neighbours: 25; t-distributed stochastic neighbor embedding-tSNE-based embedding). **c** | The distribution of the corresponding Areas Under (AU) the- ROC (AUROC) curve values (future TB-IRIS versus no TB-IRIS) across the full range of TPNN values (5-50, NN: 25, n=25 subjects). Shading represents 95% Confidence Intervals (CI). Source data are provided as a Source Data file.

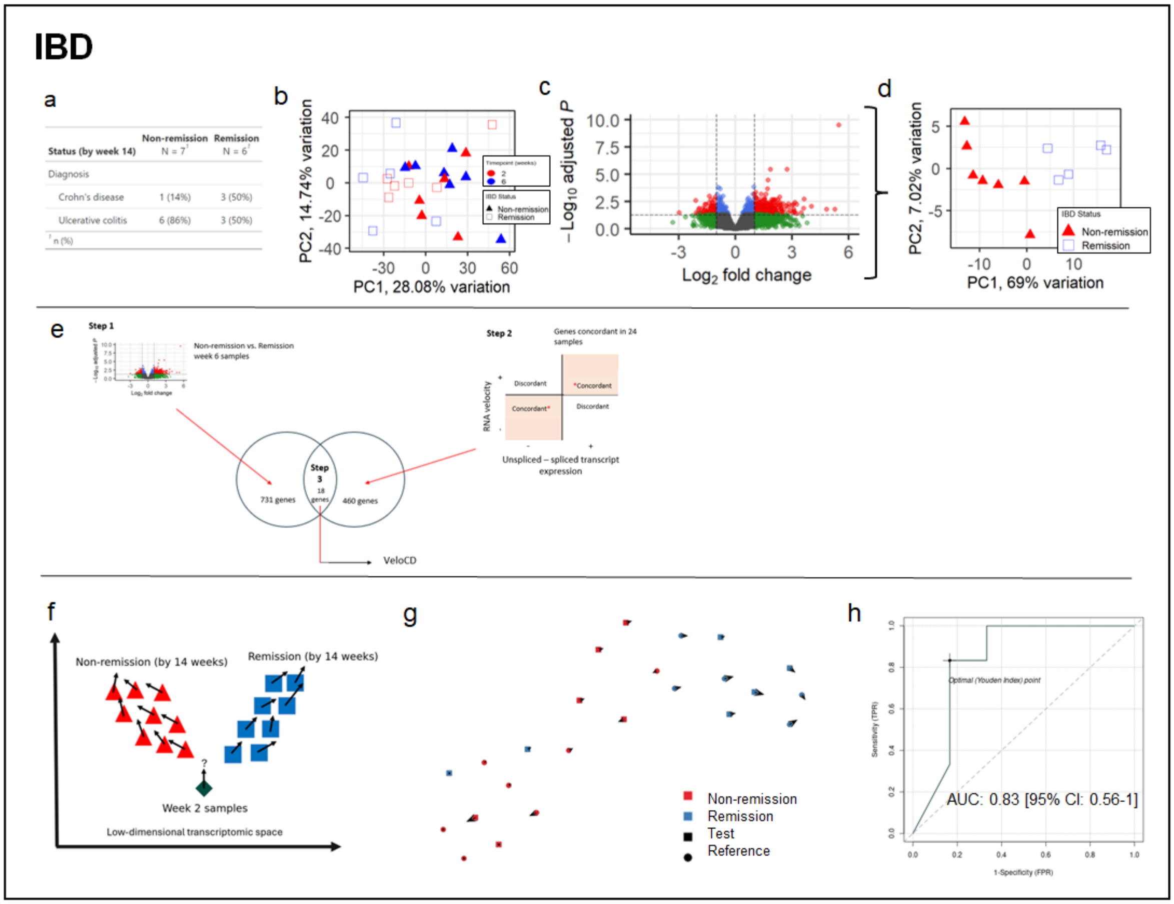

Supplementary Fig. 15: RNA velocity analysis of treatment response in inflammatory bowel disease (IBD).

a | Characteristics of study subjects. One subject who achieved remission did not have a week 6 sample and one subject who did not achieve remission did not have a week 2 sample. **b** | Principal Component Analysis (PCA) plot of the spliced transcript expression (20,377 genes, n=24 samples) of all expressed genes. Samples colored by timepoint after starting infliximab therapy (week 2 red, week 6 blue) and shaped by clinically defined remission status at week 14 (non-remission, triangle; remission, square). **c** | Volcano plot showing the differentially expressed genes (DEGs, red and blue) at week 6 between those who did (n=5) and did not go into remission by week 14 (n=7). Dots represent individual genes; red, statistically significant DEGs with absolute $\log_2$ fold change (FC)>1; green, non-significant genes with absolute $\log_2$FC>1; blue, statistically significant DEGs with absolute $\log_2$FC≤1; grey, non-significant genes with absolute $\log_2$FC≤1. **d** | PCA plot of the spliced transcript expression of the significant genes from the volcano plot (414 genes) in week 6 samples (n=12 in total, n=5 remission, n=7 non-remission). **e** | Schematic showing the gene selection framework. **f** | Schematic showing the drop-one-in approach applied to the IBD dataset. **g** | RNA velocity fate map of all 24 samples generated from an 18 gene signature (number of neighbours: 11, transition probability number of neighbours: 23, Uniform Manifold Approximation and Projection-UMAP-based embedding). Sample points are coloured by remission status (red: remission, blue: non-remission) and shaped by cohort (test, squares; reference, circles) **h** | ROC curve showing the performance VeloCD using the 18 gene signature to predict future remission (n=5) versus non-remission (n=7) from week 2 samples (transition probability number of neighbours: 4, number of neighbours: 9, 2-dimensional UMAP-embedding method). Other abbreviations: PC (Principal Component). Source data are provided as a Source Data file.

Supplementary Table 3: The 18-gene Inflammatory Bowel Disease (IBD) signature.

Ensembl ID	Gene Symbol
ENSG00000173120	*KDM2A*
ENSG00000033327	*GAB2*
ENSG00000165410	*CFL2*
ENSG00000103381	*CPPED1*
ENSG00000135709	*KIAA0513*
ENSG00000108306	*FBXL20*
ENSG00000168610	*STAT3*
ENSG00000268833	NA
ENSG00000280721	*LINC01943*
ENSG00000101236	*RNF24*
ENSG00000158470	*B4GALT5*
ENSG00000185808	*PIGP*
ENSG00000189366	*ALG1L1P*
ENSG00000135083	*CCNJL*
ENSG00000112667	*DNPH1*
ENSG00000112299	*VNN1*
ENSG00000146955	*RAB19*
ENSG00000168939	*SPRY3*

NA = Gene symbols unavailable

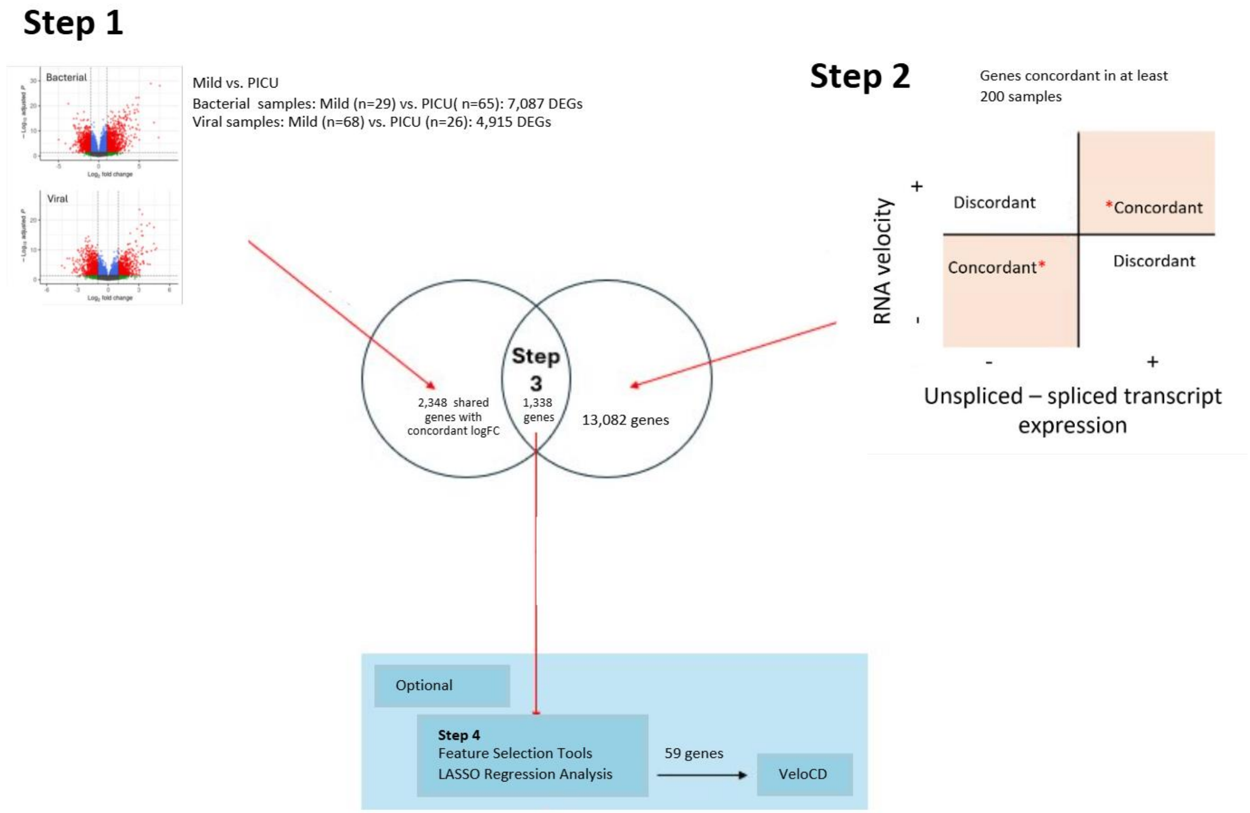

Supplementary Fig. 16: The application of the gene selection framework on the Personalized Risk assessment in Febrile illness to Optimize Real-life Management (PERFORM) RNA-Seq dataset.

Step 1 illustrates the results of the differential expression analysis between the mild and Pediatric Intensive Care Unit (PICU)-recruited severe illness children stratified by diagnosis (bacterial or viral). Step 2 illustrates the Discordant-Concordant analysis of this dataset, which compared the direction of the RNA velocity and unspliced minus spliced (transcript expression). Step 3 shows the number of genes intersecting between these two analyses, which was then input into Least Absolute Shrinkage and Selection Operator (LASSO) regression analysis. The gene signature from the latter was then used for RNA velocity analysis.

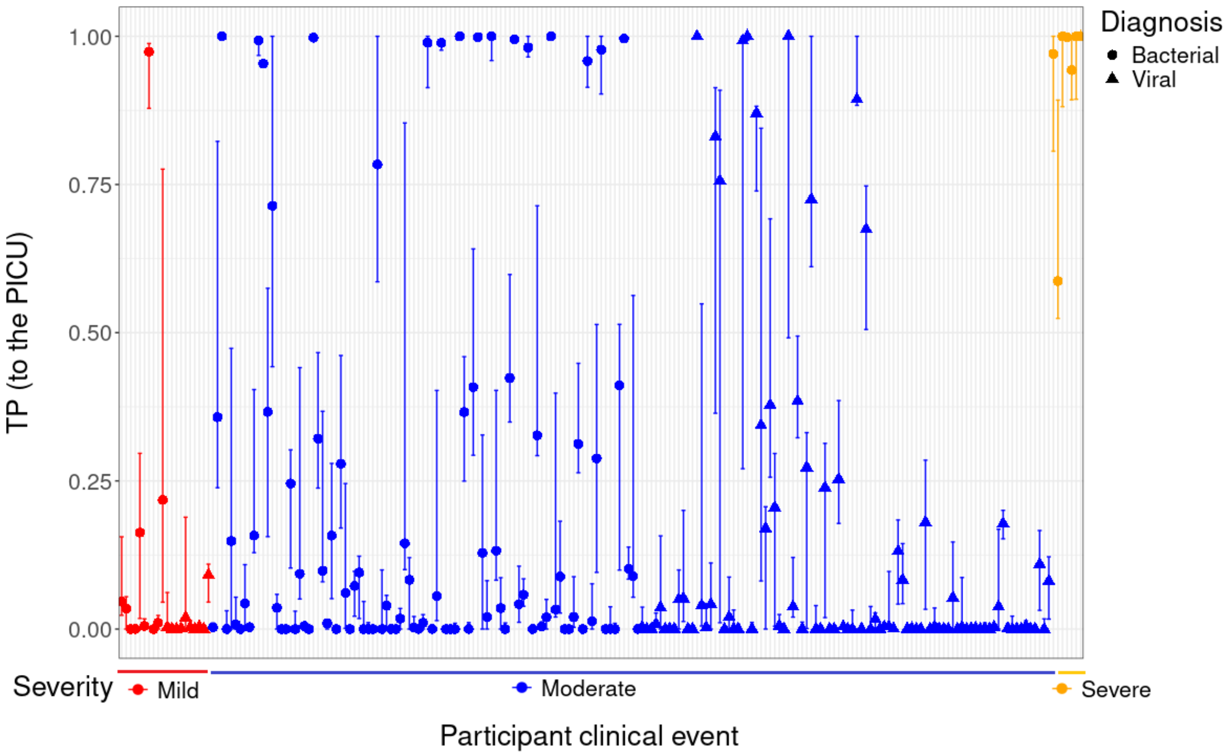

Supplementary Fig. 17: Distribution of transition probability values for progression to severe illness for each test subject in the Personalized Risk assessment in Febrile illness to Optimize Real-life Management (PEFORM) RNA-Seq dataset.

Lower and upper range, and median Transition Probability (TP) value for transition to the severe group (admission to the Paediatric Intensive Care Unit-PICU) are shown for each subject in the test set (n=211) across the full range of TP-Number of Neighbours (NN, TPNN) value runs, stratified by actual maximum severity of illness. Sample points are coloured by the severity and shaped by the diagnosis of the patient. Source data are provided as a Source Data file.

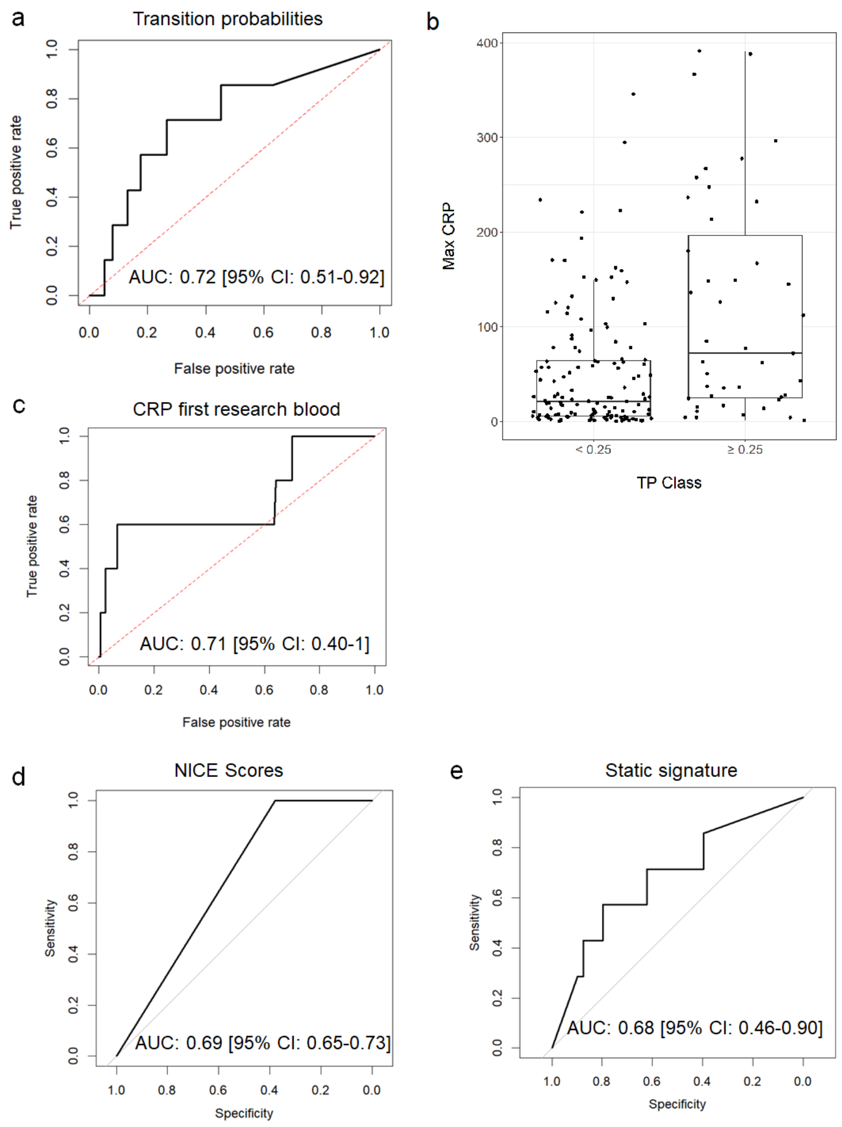

Supplementary Fig. 18: A comparison of the predictive accuracy of RNA velocity-based transition probabilities versus C-Reaction Protein (CRP) measured at first blood, National Institute of Health and Care Excellence (NICE) scores, and the 50-gene static gene signature.

a | Receiver Operating Characteristic (ROC) curve and corresponding Areas Under (AU) the ROC (AUROC) curve value for the transition probabilities (TPs, from RNA velocity) to the PICU (samples classified by whether they later transferred to the PICU or not, n=184). **b**| The distribution of the maximum CRP values during illness for each moderate clinical event with these values available (n=180) in the Febrile illness to Optimize Real-life Management (PERFORM) cohort (TP group composition <0.25, n=137; ≥0.25, n=43). The lower and upper hinges of each boxplot represent the 25th and 75th quartiles of each set of samples. The middle line represents the median. The length of the whiskers represents 1.5 times the inter-quartile range. **c-e**| Additional ROC curves for CRP measured at the first blood sample (later transition to PICU or not) (**c**), NICE risk categories (**d**), and a 50-gene static gene signature (**e**) demonstrating performance to distinguish PERFORM subjects who later transitioned from the ward to Pediatric Intensive Care Unit (PICU; n=7 subjects for **a**, **d** and **e;** n=5 for **c**) versus those who did not (n=177 subjects for **a**, **d** and **e;** n=167 subjects for **c**). Other abbreviations: Confidence Interval (CI). Source data are provided as a Source Data file.

Supplementary Table 4: The association between clinical features and transition probabilities in the Febrile illness to Optimize Real-life Management (PERFORM) subjects with moderate illness at presentation.

Clinical Feature	TP ≥ 0.25<br>n (%)	TP < 0.25<br>n (%)	OR	95% Confidence Interval	p-value
On-presentation					
Sex					
Female	19 (44.19)	62 (43.97)			
Male	24 (55.81)	79 (56.03)	1.01	0.48, 2.12	1.00
Triage Code					
Very Urgent	27 (69.23)	79 (59.40)			
Standard	12 (30.77)	54 (40.60)	1.53	0.68, 3.63	0.35
Ill Appearance					
Yes	19 (51.35)	60 (47.24)			
No	18 (48.65)	67 (52.76)	1.18	0.53, 2.62	0.71
Life Saving Intervention					
Yes	4 (9.30)	13 (9.42)			
No	39 (90.70)	125 (90.58)	0.99	0.22, 4.44	1.00
Phenotype					
Bacterial	28 (65.12)	66 (46.81)			
Viral	15 (34.88)	75 (53.19)	2.11	0.99, 4.65	0.038
Future Severity					
Surgery Performed					
Yes	11 (25.58)	11 (7.80)			
No	32 (74.42)	130 (92.20)	4.02	1.44, 11.28	0.0052
Transfer to the PICU					
Yes	4 (9.30)	3 (2.13)			
No	39 (90.70)	138 (97.87)	4.67	0.76, 33.18	0.053

Odds Ratios (OR) were calculated using Fisher's exact test, for being in the female, bacterial and more severe phenotypic groups. Values are given to two decimal places. Rows with 95% confidence intervals used Fisher's exact test (two-sided). All subjects in this analysis were recruited in the ED and admitted to the ward, n=184. Other abbreviations: Transition Probability (TP), Pediatric Intensive Care Unit (PICU).

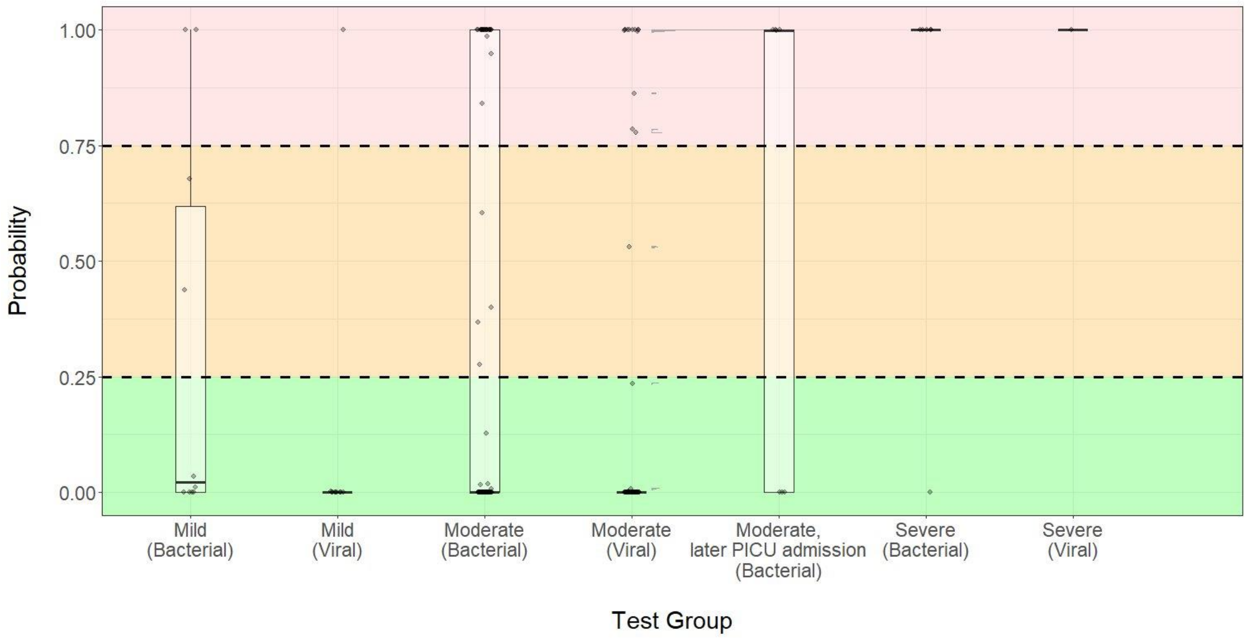

Supplementary Fig. 19: Probabilities of transition to Pediatric Intensive Care Unit (PICU) using a static 50-gene signature in Febrile illness to Optimize Real-life Management (PERFORM) test subjects (n=211).

The predicted probability for each test set subject (n=10 mild bacterial, n=10 mild viral, n=1 severe viral, n=6 severe bacterial, n=94 moderate bacterial, n=90 moderate viral subjects) to transition to the PICU. The lower and upper hinges of each boxplot represent the 25th and 75th quartiles of each set of samples. The middle line represents the median. The length of the whiskers represents 1.5 multiplied by the inter-quartile range. Shading represents different transition probability regions (green: 0-0.25, orange: 0.25-0.75, red: 0.75-1). Source data are provided as a Source Data file.

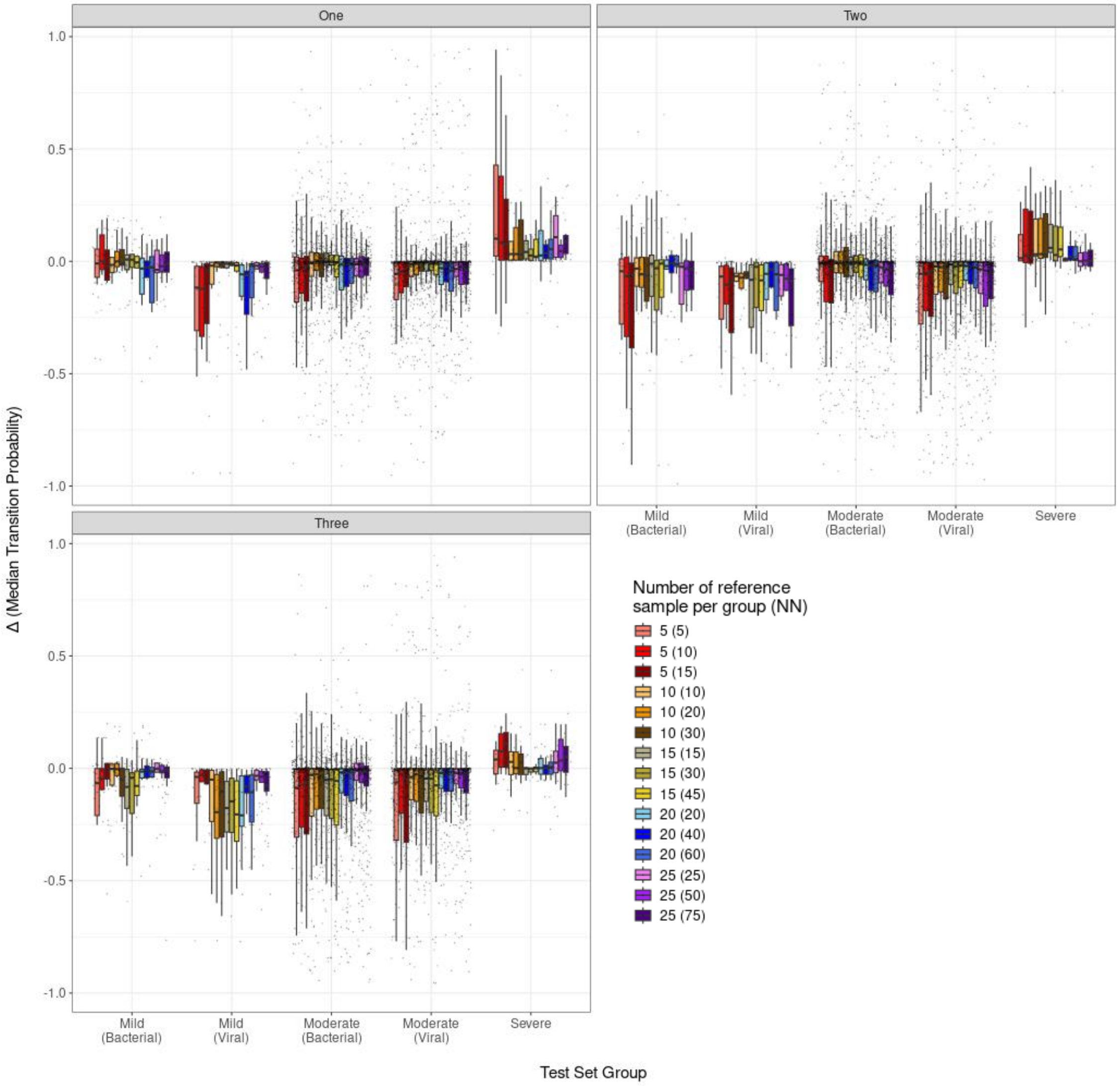

Supplementary Fig. 20: The effect of reference set sample size and composition on transition probabilities in the Febrile illness to Optimize Real-life Management (PERFORM) test cohort.

Each set of boxplots represents three replicate sets of runs (One, Two and Three) with independently randomly selected sets of reference samples (overlap between reference sample sets allowed). RNA velocity analysis was performed for each PERFORM test subject (n=211 of which, n=10 mild bacterial, n=10 mild viral, n=1 severe viral, n=6 severe bacterial, n=94 moderate bacterial, n=90 moderate viral subjects) using 5, 10, 15, 20 or 25 reference samples per diagnostic and severity group (4 groups: mild bacterial, mild vial, severe bacterial, severe viral; total reference set sizes, n=20, 40, 60, 80 or 100). Each reference set was run across three Number of Neighbour (NN) values specified by the number in the brackets on the key. Median transition probabilities were calculated for each NN sample run for each NN across a range of Transition Probability Number of Neighbours (TPNN) values (ranges: 2-19, 2-39. 2-59, 2-79, 2-99). The median transition probability values were then subtracted from the corresponding sample's transition probability at the corresponding TPNN value calculated using the full set of reference samples (delta median transition probability, n=188). The reference sample size (per group) and NN values are represented by the colors of each boxplot with test-set subjects segregated by severity and aetiology of infection. The lower and upper hinges of each boxplot represent the 25th and 75th quartiles of each set of samples. The middle line of each boxplot represents the

median. The length of the whiskers represents 1.5 multiplied by the inter-quartile range. Source data are provided as a Source Data file.

Supplementary Table 5: Participating recruitment sites and ethical approvals for the PERFORM study

Country	Site	Ethical Approval Reference
Spain	Hospital Clínico Universitario de Santiago de Compostela	2016/331
United Kingdom	St Mary's Hospital, Imperial College Healthcare NHS Trust, London<br>Alder Hey Children's Hospital, Liverpool<br>Great North Children's Hospital, Newcastle upon-<br>Tyne<br>John Radcliffe Hospital, Oxford<br>Royal Alexandra Children's Hospital, Brighton	16/LO/1684
Netherlands	Sophia's Children's Hospital, Rotterdam<br>Academic Medical Centre, Amsterdam<br>Radboud UMC, Nijmegen	NL58103.091.16
Greece	P. and A. Kyriakou Children's Hospital, Athens	415/13.06.16
Austria	Medizinische Universität Graz, Graz	28-518 ex 15/16
Slovenia	University Medical Centre Ljubljana	0120-483/2016-3
Latvia	Children's Clinical University Hospital, Riga	1/16-07-14
Germany	Dr. von Hauner Children's Hospital, Ludwig-Maximilians-University, Munich	699-16
Switzerland	University Children's Hospital, Universität Bern, Bern	2016-01835
