# Supplementary material for: Predicting trajectories of illness using RNA velocity of whole blood
Source: Nat Commun. 2026 May 6;17:3652. doi: 10.1038/s41467-026-71685-5 (PMC13149583; doi:10.1038/s41467-026-71685-5)
Supplement: Supplementary file 2 — Description of Additional Supplementary Files [file 41467_2026_71685_MOESM2_ESM.pdf]

Description of Additional supplementary files

Supplementary Data 1: Gene signature tables, NA denotes unavailable gene symbols.

Supplementary Data 2: Gene Ontology analysis.
